# Differential cellular gene expression in duck trachea infected with a highly or low pathogenic H5N1 avian influenza virus

**DOI:** 10.1186/1743-422X-10-279

**Published:** 2013-09-10

**Authors:** Pascale Massin, Claire Deleage, Aurélie Oger, François-Xavier Briand, Hélène Quenault, Yannick Blanchard

**Affiliations:** 1 Anses Ploufragan/Plouzané laboratory, Virologie Immunologie Parasitologie Aviaires et Cunicoles, B.P. 53, Ploufragan 22440, France; 2 Anses Ploufragan/Plouzané laboratory, Génétique Virale et Biosécurité, B.P. 53, Ploufragan, 22440, France; 3 European University of Brittany, Rennes, France

**Keywords:** Avian influenza virus, Highly pathogenic influenza, Low pathogenic influenza, H5N1, Suppressive subtraction hybridisation, Microarray, Muscovy duck, Trachea, Host-pathogen interactions

## Abstract

**Background:**

Avian influenza A (AI) viruses of subtypes H5 can cause serious disease outbreaks in poultry including panzootic due to H5N1 highly pathogenic (HP) viruses. These viruses are a threat not only for animal health but also public health due to their zoonotic potential. The domestic duck plays a major role in the epidemiological cycle of influenza virus subtypes H5 but little is known concerning host/pathogen interactions during influenza infection in duck species. In this study, a subtracted library from duck trachea (a primary site of influenza virus infection) was constructed to analyse and compare the host response after a highly or low pathogenic (LP) H5N1-infection.

**Results:**

Here, we show that more than 200 different genes were differentially expressed in infected duck trachea to a significant degree. In addition, significant differentially expressed genes between LPAI- and HPAI-infected tracheas were observed. Gene ontology annotation was used and specific signalling pathways were identified. These pathways were different for LPAI and HPAI-infected tracheas, except for the CXCR4 signalling pathway which is implicated in immune response. A different modulation of genes in the CXCR4 signalling pathway and TRIM33 was induced in duck tracheas infected with a HPAI- or a LPAI-H5N1.

**Conclusion:**

First, this study indicates that Suppressive Subtractive Hybridization (SSH) is an alternative approach to gain insights into the pathogenesis of influenza infection in ducks. Secondly, the results indicate that cellular gene expression in the duck trachea was differently modulated after infection with a LPAI-H5N1 or after infection with a HPAI-H5N1 virus. Such difference found in infected trachea, a primary infection site, could precede continuation of infection and could explain appearance of respiratory symptoms or not.

## Background

The Influenza A virus genus is divided into subtypes based on the combination of two surface glycoproteins: hemagglutinin (HA, 16 subtypes) and neuraminidase (NA, 9 subtypes) [1]. The subtypes H5 and H7 of avian influenza A (AI) viruses can be both further divided into two groups of high or low pathogenic influenza A viruses (HPAI or LPAI, respectively) [2]. LPAI viruses induce mild or no symptoms in domestic ducks but replicate massively into the intestinal tract allowing the release of high titres of virus into faeces. In contrast, H5-HPAI viruses induce various clinical signs ranging from asymptomatic respiratory and digestive tract infections to systemic and severe symptoms leading to fatal outcome depending on age and duck species [3]. AI viruses have caused several serious epizootics within poultry, in particular the HPAI H5N1 virus which propagated between 2003 and 2006 from Asia to Europe and Africa, and caused the death of millions of chickens and other poultry leading to massive economic loss [4,5]. Indeed, poultries are key intermediates in the transmission of AI from wild avian species to mammalian species such as pigs and humans. The H5 virus is now enzootic in several Asian countries and represents one major concern for animal health but also for public health due to its zoonotic and pandemic potential. Indeed HPAI H5N1 has been transmitted from poultry to humans and was responsible for the death of 374 people as reported by WHO the 26th April 2013 [6,7].

Various studies have been performed to better characterize host/pathogen interactions between mammals and influenza A viruses and to identify key genes or pathways implicated in the virus pathogenicity and host response to infection [8-13]. Virus-host interaction investigations have been particularly boosted by the development of the microarray technology in many mammalian models [14-20], for which microarray tools, gene annotation and signalling pathways description are abundant. In contrast information concerning the molecular pathogenesis of AIV and the regulation of host gene response after AIV infection in avian species is scarce and essentially performed in chickens. The recent release of the chicken genome is a great help for scientists to investigate mechanisms involved in host response to the infection in chicken and the appropriate microarrays have been developed recently and used in various virus infection studies [21-30]. Concerning duck species, no microarrays are available and molecular genetics tools have made their first steps recently [31].

Some studies have described the difference of pathobiology between a LPAI and a HPAI infection in various ducks species but to our knowledge, only three studies have compared the response of ducks following LPAI or HPAI infection, focusing on immune response only [32-34]. In these studies, the authors concluded that a difference of immune response, specific for the virus and the infected tissue (lung or intestine), might explain the difference of pathogenicity between LPAI and HPAI infection in Pekin ducks. A few other studies have been performed to compare the immune response of Pekin, Mallard and Muscovy ducks and provide evidences of some differences that might account for the variability of HPAI pathogenesis in between duck species [34-37]. A limitation of these comparative studies is their focus on a very limited number of genes, usually selected on the basis of published data in other species for which the symptoms and outcome of an AI infection might be different from those observed in duck species. Therefore, these studies provide a very limited amount of information which is not necessarily the most relevant for a duck model, and thus does not give an objective overview of host/pathogen interactions between duck, cells or tissues, and influenza A virus.

For a better understanding of the host/pathogen interactions between H5N1 viruses and domestic ducks and with the aim of giving an overview of host/pathogen interactions between duck respiratory tract and HP- as compared to LP-influenza A virus, we examined and compared the mRNA expression of genes into a primary infection site, the trachea, after infection with a HPAI or a LPAI H5N1. To overcome the fact that there was no release of the duck genome and no duck specific microarray, we focused on only differentially expressed genes by creating subtracted libraries from duck trachea and used them to set up a duck microarray for analysis of HPAI and LPAI H5N1 infection.

## Results

### Validation of the experimental infection model

Tracheal explants were prepared as described in Materials and methods and infected with 200 μl of 2×10^7^ TCID_50_/ml of HPAI virus per trachea, the same dose as the one used for library construction. At 24 h p.i., the ciliary beats were dramatically reduced to up to 30% as compared to mock-infected tracheas (100% ciliary beats) attesting for an efficient infection of the tracheas. To verify that infection occurred all along tracheas at 24 h p.i., LPAI-infected tracheas were cut into 3 parts and subjected to RNA extraction and to amplification of influenza matrix (M) segment using real-time RT-PCR as described in Materials and methods. Levels of copy numbers were similar for the three parts of trachea at 24 h p.i. (4 to 8.10^7^ M copy per μl) assessing for a homogeneous contact of the inoculum with the whole trachea. In addition, RNA of LPAI- and HPAI-infected tracheas used for the preparation of probes were also subjected to amplification of influenza matrix M segment using real-time RT-PCR and we did not observe significant difference.

### Construction of specific duck trachea subtracted libraries

Four subtracted libraries were constructed using entire tracheal explants from SPF Muscovy ducks, infected or not with a French HPAI H5N1 strain belonging to clade 2.2.1 and subjected to the subtractive suppression hybridisation (SSH) procedure: two viral-induced and two viral-repressed cDNA libraries corresponding to sequences induced and repressed, respectively, at 4 h or 8 h p.i. From these 4 libraries, 1141 individual bacterial clones were randomly isolated for the two viral-induced libraries and 950 bacterial clones for the two viral-repressed libraries representing around 10% of all clones obtained. Those 2091 clones were used to construct our duck specific microarray as described in the Materials and methods section.

**Figure 1:**
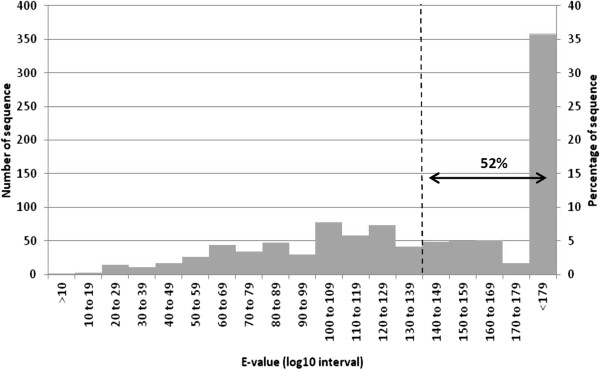
**Distribution of sequences depending on E-value result.** For each E-value, number of obtained sequences was counted and was represented into histogram in order to see the repartition and the relevance of gene identification. More than 50% of sequences obtained an E-value below 10^-140^.

The 2091 clones were subjected to sequencing as described in the Materials and methods section. For 1013 sequences out of 2091, a relevant blast result was obtained. For the 1078 remaining sequences we did not obtain a significant blast result due to either poor sequence quality or the absence of positive match in Genbank library. The e-values distribution of relevant blast results shown that 52% of sequences were above 10^-140^, supporting an accurate identification of the genes (Figure 1). 114 sequences had good homology with avian mitochondrial genes and 115 sequences had homology with avian BAC clones or complete avian cDNA of putative protein of unknown function. 784 sequences have been clearly identified, to date, as avian genes and corresponding to 210 different genes. These genes belong to several different functional families: cell cycle, metabolism, immune response, cytoskeleton network.

### Statistical analysis of microarray results

In order to compare the HPAI and LPAI infections in the trachea using our microarray, new tracheal explants were infected with either strains (HPAI or LPAI) and RNA were extracted and processed for microarray experiment as described in Materials and methods section. Sixteen microarrays were hybridised allowing four replicates for each experimental condition: 4 microarrays for each time point of the experiment (4 and 8 h p.i.) and for each strain (LPAI and HPAI).

**Figure 2:**
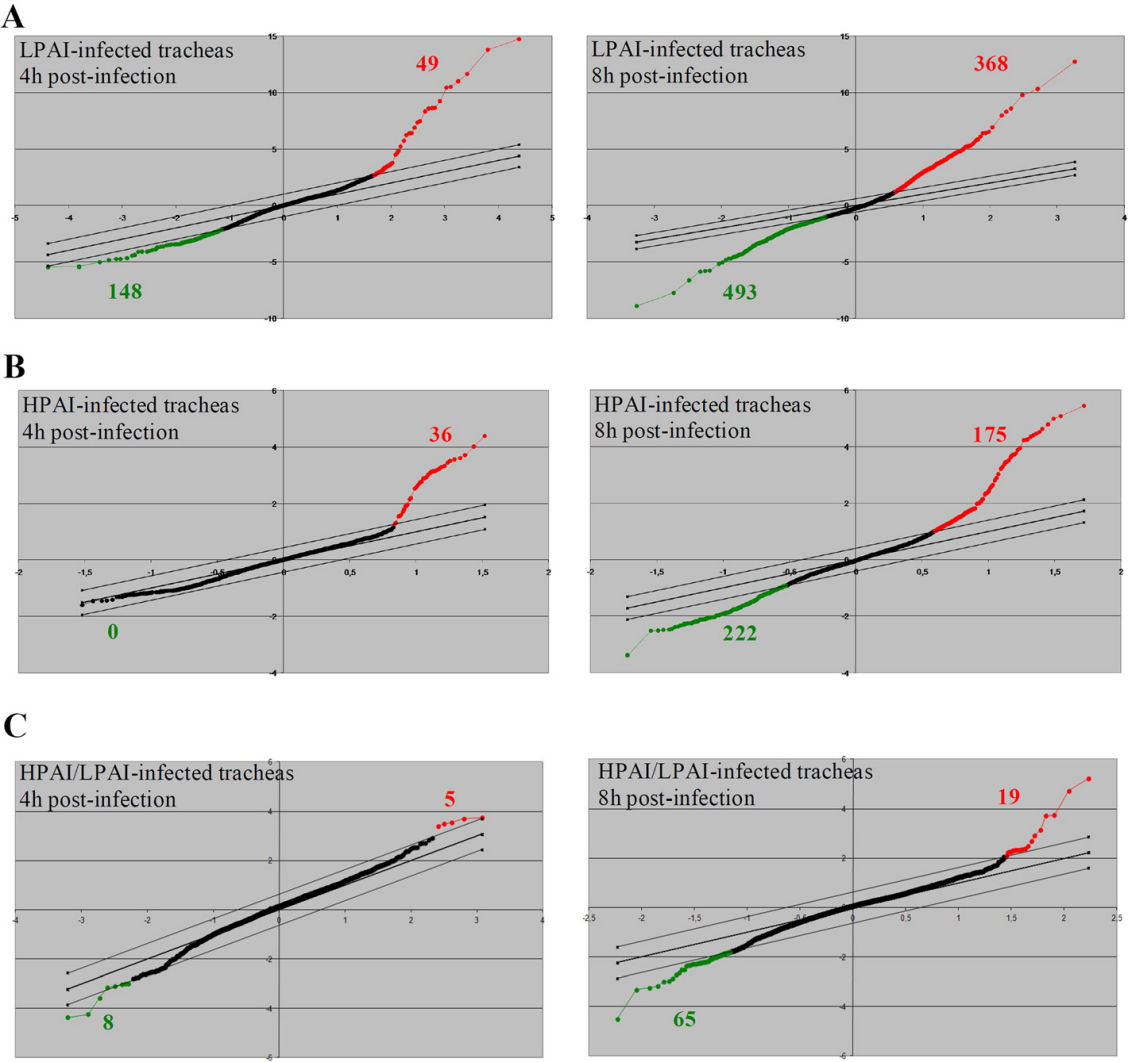
**Plots of sequences differentially expressed in duck trachea after H5N1 infection using the significance analysis micro-array software. A** and **B**: One-class analyses of microarray data were performed for each dataset: after LPAI-H5N1 **(A)** or HPAI-H5N1 **(B)** at 4 h (left panels) or 8 h (right panels) post-infection, as compared to mock-infected. The false discovery rate was set at 4.76 (**A**, left panel), 4.81 (**A**, right panel), 5.7 (**B**, left panel) and 4.82% (**B**, right panel). **C**: Two-class analyses of microarray data were performed for each time post-infection: at 4 h (right panel) or 8 h (left panel) post-infection by comparing HPAI-H5N1 infection to LPAI-H5N1 infection. The false discovery rate was set at 0 (**C**, right panel) and 4.15% (**C**, left panel). The x-axis values represent expected expression and the y-axis represent observed expression. Parallel lines represent the threshold with the corresponding false discovery rate set. Black dots represent sequences not differentially expressed between the two compared conditions. Red dots and red numbers represent significant up-regulated sequences in HPAI- or LPAI-infected tracheas as compared to mock-infected **(A, B)**, or HPAI-infected tracheas as compared to LPAI-infected tracheas **(C)**. Green dots and numbers represent significant down-regulated sequences in HPAI- or LPAI-infected tracheas as compared to mock-infected **(A, B)**, or HPAI-infected tracheas as compared to LPAI-infected tracheas **(C)**.

After normalisation of the raw data, we performed a first statistical analysis by comparing signals obtained with probes corresponding to infected tracheas (HPAI- or LPAI-infected) versus signals obtained with probes corresponding to mock-infected tracheas. For this purpose, SAM software was used and results were considered for a calculated false discovery rate (FDR) of 5%. SAM plot results are presented in Figure 2A and B. At 4 h p.i., 49 spots were up-regulated and 148 spots were down-regulated in LPAI-infected tracheas whereas 36 spots were up-regulated but 0 down regulated genes in HPAI-infected tracheas (Figure 2A and B, left panel). At 8 h p.i., number of differentially expressed sequences significantly increased (Figure 2A and B, right panel). In LPAI-infected tracheas, 368 sequences were up-regulated and 493 sequences were down-regulated. In HPAI-infected tracheas, 175 sequences were up-regulated and 222 were down-regulated.

This first SAM analysis gave us the difference in between infected samples compared to mock-infected ones. In a second approach, we performed a statistical analysis comparing the signals obtained with probes from HPAI-infected tracheas to signals obtained with probes from LPAI-infected tracheas. Such a comparison was possible as the reference sample used for the baseline was generated with the same pool of mock-infected duck mRNA. Results are presented in Figure 2C. A FDR fixed around 5% gave a very low number of significant differentially expressed spots between HPAI and LPAI samples: 8 down-regulated and 5 up-regulated at 4 h p.i. and 65 down-regulated and 19 up-regulated at 8 h p.i. for HPAI as compared to LPAI.

### Functional characterisation of differentially expressed genes

In order to further characterize differentially expressed sequences, sequencing data and microarray results were cross-analysed using Gene Ontology annotations with the Ingenuity Pathway Analysis software. Due to the poverty of bibliographic information for the chicken annotated genome and the duck genome, comparison was made by analogy to the orthologous genes annotation from the well-documented and annotated human, mouse and rat genomes.

**Figure 3:**
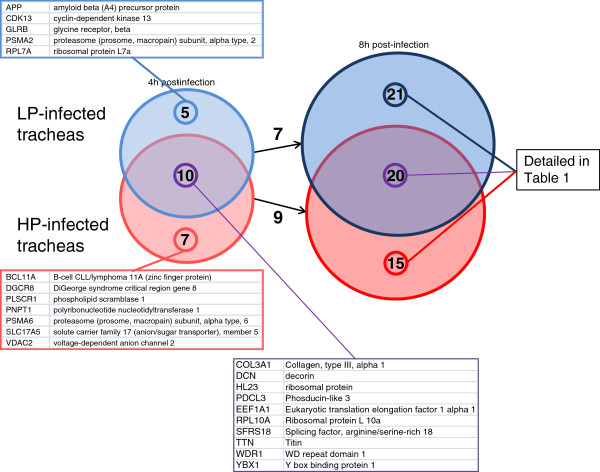
**Schematic representation of genes significantly differentially expressed during infection time course using the ingenuity pathway analysis software.** At 4 h post-infection (left circles) or 8 h post-infection (right circles), genes differentially expressed in LPAI-infected tracheas (blue circles) were compared to those differentially expressed in HPAI-infected tracheas (red circles). Common genes differentially expressed in LPAI- and HPAI-infected tracheas are represented by the junction of the two circles. Common genes differentially expressed at 4 h and 8 h post-infection are represented by the arrow between two circles. Gene lists were provided in the figure for 4 h post-infection and in Additional file 1: Table S1 for 8 h post-infection.

Among the 210 genes identified from our subtracted libraries, only 158 were referenced in Ingenuity database for analysis, the remaining 52 genes are predicted genes with no relevant annotation in the database. Trachea responses, *i.e.* differentially expressed genes in HPAI- and LPAI-infected tracheas, were compared for each time post-infection. Results are presented in Figure 3. At 4 h post-infection, trachea responses to infection were slightly different with 10 genes shared and 5 and 7 genes implicated only in LPAI- or HPAI-infection, respectively. Within those genes which appeared to be implicated only in LPAI- or HPAI-H5N1 infected tracheas, some genes are in fact implicated in the same protein complex (for example 20S-proteasome with PSMA2 and PSMA6). At 8 h post-infection, trachea responses were more different between LPAI- and HPAI-infection but some genes have potential similar functions (Additional file 1: Table S1, for example ribosomal protein L10a, L7a and LP2). Only few genes were found to be differentially expressed both at 4 h and at 8 h p.i. (7 for LPAI-infected tracheas and 9 for HPAI-infected tracheas, Figure 3).

Using the Ingenuity Pathway Analysis software, interaction networks between selected genes were inferred based on the known direct or indirect relation between these genes stored in Ingenuity’s database (related to literature). In a first time the analysis was conducted on the genes obtained after comparison to mock-infected sample. For LPAI-infected tracheas, 5 gene interaction networks can be identified and three of these networks were connected together by one or two genes. For HPAI-infected tracheas, 5 gene interaction networks were also identified and only two of these networks interacted together by one gene, and the three others were not connected to another one. Induced and repressed genes by HPAI- or LPAI-infection included in interaction networks are presented in Additional file 1: Table S1.

**Figure 4:**
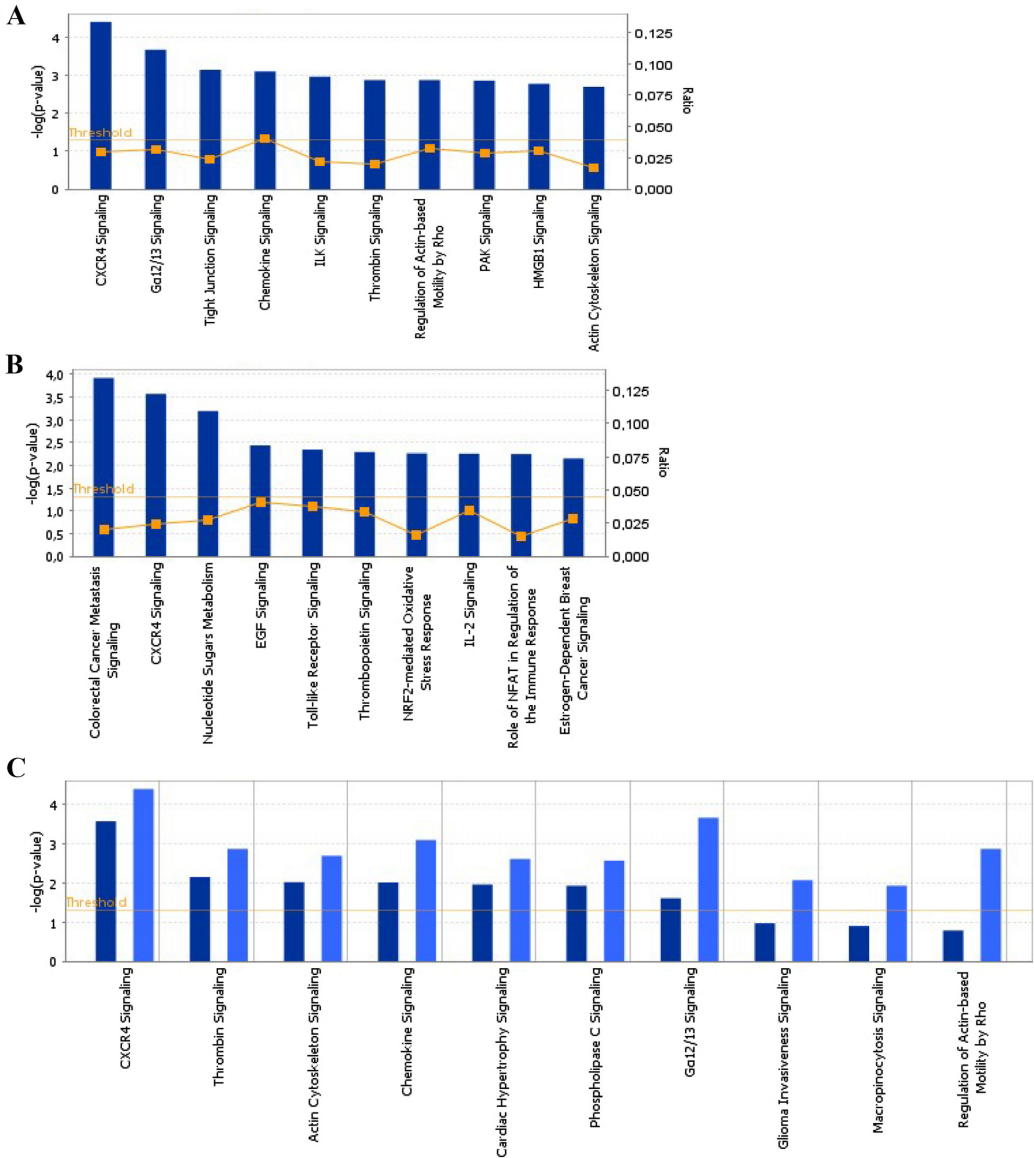
**Analysis of significant differentially expressed genes into pathway. A**: in LPAI-H5N1 infected tracheas as compared to mock-infected tracheas, **B**: in HPAI-H5N1 infected tracheas as compared to mock-infected tracheas, **C**: in HPAI-H5N1 infected tracheas as compared to LPAI-H5N1 tracheas. The ten most significant pathways are shown in graph A and B, and only the first ten common pathways between HPAI- and LPAI-infection are shown in graph C (dark blue for HPAI-H5N1 and light blue for LPAI-H5N1). The Ingenuity Pathway Analysis software was used to organise significant differentially expressed genes into different signalling pathways in which they are involved and according to the calculated –log (p-values). Data significance is represented by ratio and p-value. Ratios (yellow lines) represent the part of differentially expressed genes from a pathway related to the total number of genes for this same pathway. The p-value is calculated using a right-tailed Fisher’s exact test and corresponds to the probability that the association between genes and a given pathway is not due to random chance. The threshold corresponds to a limit of significance set by the software (p < 0.05).

Analysis of the microarray results for the signalling pathways highlighted different pathways in between LPAI- and HPAI-infected tracheas, with only the CXCR4 pathway present for both infections (Figure 4A and B). For this pathway, H-Ras, MLC and FOS were significantly modulated by both LPAI- and HPAI-infection; Rho and Gbeta were significantly modulated only by LPAI- or HPAI-infection, respectively.

In a second time, the genes selected from the comparative analysis of trachea responses after LPAI-infection or HPAI-infection were submitted to Ingenuity analysis. Only two networks, not interconnected to each other, could be identified. Repressed genes by HP-infection as compared to LP-infection included in interaction networks are presented in Additional file 2: Table S2. No induced genes were identified. Interestingly, despite the CXCR4 signalling pathway was highlighted for both HP and LP infection when compared to mock-infected samples, it was also the signalling pathway that discriminated the HP and LP infections when compared to each other. When looking at the other 9 common signalling pathways out of 10, five were previously identified as modified into LPAI-infected trachea, and none for HPAI-infection (Figure 4C).

### Validation of differential expression of genes by quantitative PCR

Differential duck tracheal gene expression after LPAI- or HPAI-infection was assessed for a selected set of genes by real-time PCRs. This set was constituted by various modulated genes *i.e.* induced or repressed genes either in HP or in LP infection. As shown in Table 1, quantitative PCR confirmed only for a limited number of genes the result obtained into microarray data analysis (SAM analysis) but not for the other. In particular, at 4 h p.i., we observed high variations in gene expression between the different samples from the same experimental condition (LPAI- or HPAI-infected tracheas) which resulted in not-statistical significant gene expression variations. Indeed, Only H-Ras, DDX3X and DCN were found statistically significantly down-regulated in HPAI-infected tracheas as compared to LPAI-infected tracheas. At 8 h p.i., we obtained more statistical significant results: TRIM33 and FOS were up-regulated whereas H-Ras, EEF1A1 and DDX3X were down-regulated in HPAI-infected tracheas as compared to LPAI-infected tracheas.

**Table 1:** Relative amount of differentially expressed genes in H5N1-infected tracheas as compared to control and in HPAI-H5N1- as compared to LPAI-H5N1 infected tracheas using quantitative PCR

**Identified gene**	**Genbank accession number**	**Fold change at 4 h p.i.**			**Fold change at 8 h p.i**		
		**LP versus control**^**a**^	**HP versus control**^**a**^	**HP versus LP**^**b**^	**LP versus control**^**a**^	**HP versus control**^**a**^	**HP versus LP**^**b**^
TRIM33	XM_418009	0.50±0.21	1.41±1.34	2.84	0.69±0.24	1.19±0.15	1.72*
H-Ras	XM_415752	0.87±0.47	0.17±0.23	0.20**	0.22±0.02	0.15±0.04	0.68**
RABL5	CR406564	1.45±2.23	28.85±34.10	19.85	2.43±1.49	7.06±5.30	**2.9**
FOS	XM_002200534	3.36±2.89	2.26±1.90	0.67	0.99±0.73	3.04±0.86	3.08*
RhoA	NM_001245622	1.60±1.24	3.96±6.14	0.40	8.29±12.01	3.89±4.41	**0.47**
EEF1A1	NM_204157	2.18±1.46	0.15±0.03	**0.07**	1.31±0.17	0.59±0.25	0.45**
DDX3X	XM_002190542	1.27±0.14	0.29±0.14	0.22*	1.07±0.02	0.4±0.25	0.37**
DCN	AF125250	5.95±1.94	0.29±0.23	0.05**	0.21±0.06	0.22±0.09	1.02
IL13	AC233977	13.14±12.99	7.37±5.72	0.56	5.61±3.73	23.89±20.95	4.26

^a^Fold change is the mean ± SD of gene expression in 4 infected tracheas as compared to control.

^b^Fold change is the ratio of gene expression in HPAI-infected tracheas as compared to LPAI-infected trachea as indicated.

* p < 0.05 and ** p < 0.1 (two-sample Student t-test); bold data were two-class SAM significant.

## Discussion

Aquatic birds play an important role into the dissemination and transmission of influenza A viruses between species. However, little is known concerning host/pathogen interactions during influenza infection in these species and particularly into duck, a poorly studied species. In order to bypass the lack of information concerning duck genome, we decided the construction and use of duck trachea subtracted libraries to analyse and to compare LPAI- and HPAI-H5N1 infection.

In this study, we focused onto a primary site of infection for influenza A viruses, the trachea, for which the response to influenza infection is determining for the outcome of the infection. The *in vitro* model was optimised by using entire trachea instead of tracheal rings in order to limit a wound healing response that would interfere with the cellular response to infection. We further checked that the infection occurred homogeneously in the entire tracheas prepared whatever the virus used (LPAI or HPAI). M gene was detected by real-time RT-PCR and ciliostasis was observed all along the trachea for both the LPAI- and HPAI-H5N1 infected samples indicating that the virus infected and replicated all along the tracheas.

The efficacy of the SSH strategy for host response analysis and discovery of modulated genes in poorly-studied species, in association or not with microarray, has been well established [38-44]. The suppressive subtractive hybridisation (SSH) procedure is designed to subtract cell responses that occur in both control and infected tracheas allowing focusing only on the differences between the two sets of RNA used. Even if this strategy allowed us to generate and analyse gene datasets from duck, it however suffers some drawbacks: –First, and foremost, there is an important loss of information occurring after sequencing of our subtracted libraries with only half of the sequences generating a pertinent blast result. At the end of the process only 210 different avian genes were identified. –Secondly, several sequences corresponded to genes encoding proteins with unknown function or predicted proteins that were not annotated in databases. In these conditions the annotation based analysis, with tools like the Ingenuity Pathway Analysis software, are performed with a reduced number of sequences and *in fine* poorly informative. Another limit of this approach resides in the fact that, due to the relative paucity of the data concerning avian species in the literature, as compared to the mammalian species, our annotation based analysis assume a functional equivalence of the avian and duck genes with their mammalian (human, rat and mouse) orthologs which is not demonstrated and most probably exaggerated. However, many signaling pathways, including pathways concerning immunity, have been described and involve the same genes in various species. Despite these limitations, our subtracted libraries corresponding to induced or repressed sequences at 4 h and 8 h p.i. contained 1141 and 950 clones, respectively. Using statistical analysis, only 19% of these clones were differentially expressed. We observed that between LPAI- and HPAI-infection, there were significant differentially expressed sequences which possibly might be related to the difference of pathogenicity between LPAI and HPAI viruses. In order to identify if these sequences correspond to genes implicated in some specific mechanisms involved in influenza infection (HPAI and/or LPAI) in duck trachea, gene ontology annotations using Ingenuity Pathway Analysis software was used. Among identified pathways, our findings (*i.e.* a variation of cellular gene expression between HP- and LP-infected tracheas, are consistent with previous studies which demonstrate differential modulation of the immune response according to the AI strains [45-48]. Furthermore, modulation of the immune response throughout the direct interaction between cellular and viral proteins could inflect the outcome of AIV infection in the host (NS1 in particular, for a review see [49]).

In our study, the infection of duck tracheas with a HPAI- or a LPAI-H5N1 induced a different modulation of genes in the CXCR4 signalling pathway. The CXCR4 pathway is activated by the fixation of the stromal cell-derived factor 1 alpha (SDF-1α) on the chemokine (CXC motif) receptor 4, a G-protein-coupled receptor. This pathway play fundamental roles in distinct signalling pathways like cell migration, transcriptional activation and cell growth, and is implicated in different physiological processes (homeostatic regulation of leukocytes traffic, haematopoiesis for example). In the case of HIV-1 infection, CXCR4 has been defined as a co-receptor which, binding by the virus, mediates membrane fusion and signalling transduction that might facilitate viral infection and pathogenesis [50]. In our case we showed that, within CXCR4 signalling pathways, Gβ, H-Ras, Rho, MLC and FOS expressions were modified during early time of LPAI- and/or HPAI-infection. The expression of Gβ was up-regulated in HPAI-infected tracheas as compared to control. This Gβ protein is part of the heterotrimeric G-protein coupled to CXCR4. This activated G-protein induces transcription, cell adhesion, chemotaxis, cell survival via different signalling pathways. When comparing HPAI-infected to LPAI-infected tracheas, we found that H-Ras was significantly more induced in LPAI-infected tracheas whereas a similar down regulation was observed for FOS. H-Ras is a small GTPase belonging to the Ras oncogene superfamily, Ras subfamily impacts multiple cellular processes (cell survival, growth, differentiation) via different pathway like the Raf/Mek/Erk pathway, PI3K pathway, or RalGDS signalling pathway and is a key intermediate in the transduction of signal during the immune response.

The FOS protein, down regulated in both HP and LP infection, is a leucine zipper protein that dimerize with c-Jun protein to form the AP-1 transcription factor complex. This complex has also been implicated in various biological processes: cell proliferation, differentiation and transformation, and apoptotic cell death. Concerning infectious diseases, FOS has been described to have a positive effect on hepatitis C virus (HCV) propagation and may contribute to HCV pathogenesis [51]. Rho was only found up-regulated in LPAI-infected tracheas. Rho family proteins are small GTPases also implicated in various cell functions, and have been described to be essential for B-lymphocytes development. These proteins, members of the CXCR4 signalling pathway, differentially expressed when comparing HPAI- and LPAI-infection, play a role in different virus infection or in immune response and might have a role in the difference of influenza virulence in ducks. Thus, it can be hypothesized that the HPAI has an inhibitory effect on the immune response in trachea by an inhibition of the CXCR4 signalling pathway, as compared to the LPAI virus, and then promotes a systemic infection in Muscovy ducks by evading the host immune response.

In our study, we also found that TRIM33 was up-expressed in HPAI-infected tracheas as compared to LPAI-infected. TRIM33, also called Trancriptional Intermediary Factor 1γ (TIF1γ), has been described to display an anti-viral activity limiting early and late gene expression in a human adenovirus infection model. This antiviral activity is counteracted by the Adenoviral E4orf3 protein which triggers TIF1γ to proteasomal degradation [52]. TIF1γ is however mainly described as transcriptional corepressor acting at the chromatin level and whether or not the up regulation of TIF1γ during HPAI infection is in favour of the virus or the host remains to be determined.

Dysregulation observed in this study, between these pathways and inside these pathways could explain the difference of pathogenicity between LPAI- and HPAI-infection in duck as it has been proposed by Vanderven *et al.*[34] and in macaques [53]. In addition, we observed a high number of differentially expressed spots corresponding to protein of unknown function or not already described which would deserve further attention in the light of host pathogen interactions.

## Conclusion

In conclusion, using SSH and microarray tools we showed that cellular gene expression in the duck trachea was differently modulated after infection with a LPAI-H5N1 or after infection with a HPAI-H5N1 virus. Some different signalling pathways and some difference within similar signalling pathways seemed to be implicated into the difference between LPAI- and HPAI-infection. These differences were found in infected trachea, a primary infection site, which could precede continuation of infection and could explain appearance of respiratory symptoms or not. Such findings have to be more precisely studied when duck genes are more characterised. Although SSH is an alternative approach to get insights into the pathogenesis of influenza infection in ducks as long as duck microarrays are not available, the lack of duck genome annotations and signalling pathways hampers understanding of host-virus interaction in this species.

## Materials and methods

### Entire tracheal explants preparation, viruses and infection

Entire tracheal explants were prepared from 29 days old embryonated specific pathogen free (SPF) Muscovy duck eggs (just before hatching) and after several careful washing steps, maintained at 37°C in minimal essential medium supplemented with antibiotics until use.

Regarding the viruses strains used, A/duck/France/05066b/05 (LPAI H5N1) and A/mute swan/France/06299/06 (HPAI H5N1 belonging to clade 2.2.1) were isolated by the National Reference Laboratory for Avian Influenza and Newcastle disease at the Anses Ploufragan/Plouzané laboratory, France [54,55]. Influenza viruses were amplified at 37°C in 9-day-old SPF embryonated chicken eggs. Allantoïc fluids were collected at a maximum of 4 days after inoculation and then subjected to centrifugal clarification before use as viral stocks.

Trachea infection was performed by injecting the inoculum directly into and all along the lumen of trachea using a fine needle. Trachea was then incubated at 37°C, 2% CO_2_ in inclined plates in order to keep trachea into the medium.

### RNA extraction

At 4 and 8 h post-infection (p.i.), tracheas were washed with cold PBS and then crushed directly into Trizol LS reagent (Invitrogen) using a tissuelyser and a stainless bead. RNA extraction was performed following standard Trizol protocol instructions.

### Suppressive subtraction hybridisation for libraries construction

For libraries construction, 40 entire trachea explants were prepared and infected with a high dose (200 μl of 2×10^7^ TCID50/ml per trachea) of the HPAI H5N1 strain (2 sets of 10 tracheas) or mock-infected (2 sets of 10 tracheas).

At 4 and 8 h p.i., tracheas were washed twice in cold PBS and RNA was extracted as described above. cDNA was synthesized from 4 μg of each RNA preparation using the PCR-Select cDNA Subtraction Kit (Clontech). To compare the two populations of resulting cDNA (from infected cells and control cells), a suppressive subtraction hybridisation (SSH) assay was then performed using the PCR-Select cDNA Subtraction Kit (Clontech). According to manufacturer’s instructions and briefly, the cDNA from the tester (infected) and from driver (mock-infected) were digested with *Rsa* I restriction enzyme. The tester cDNA pool was splitted into two parts and each part was then ligated to a different cDNA adaptor. During a first hybridisation step, an excess of driver (mock-infected) was added to the two tester cDNA samples, heat-denatured (98°C 1 min30) and allowed to anneal at 68°C during 6 to 12 hours. This step allowed for an equalization of high- and low-abundance sequences and simultaneously for a significant enrichment of differentially expressed cDNA sequences. In a second hybridisation step, the two primary hybridisation tester samples were mixed without denaturation step. To further select for differentially expressed sequences, heat-denatured driver cDNA was again added to these hybrid samples. As a result, the remaining subtracted, equalized single-stranded tester cDNA re-associated to form hybrids cDNA with a different adaptor on each end. These forward-subtracted samples were then used in PCR to amplify the differentially expressed sequences using primers complementary to adaptor. PCR mixtures of forward subtractions were ligated into pGEM vector and used in transformations with competent *E. coli* (TOP10, Invitrogen). According to Clontech’s instructions, for each forward subtraction (corresponding to induced genes), reverse subtraction (corresponding to repressed genes) were also performed. These experiments resulted in the construction of four libraries: -two viral-induced libraries (4 and 8 h p.i.) and -two viral-repressed libraries (4 and 8 h p.i.). Libraries were conserved as bacterial clones at −70°C in LB/glycerol medium.

### Sequences analysis and clone identification

The four duck tracheal subtracted libraries were sequenced in order to identify corresponding genes. To this purpose, isolated bacterial colonies were grown for plasmid isolation with the Wizard SV 96 Plasmid DNA purification System (Promega). The DNA sequences of purified products were determined using ABI Prism DyeTerminator Cycle Sequencing Ready Reaction Kit (Applied Biosystems) and a primer flanking the cloning site of pGEM (M13rev or T7 primer) on an automatic DNA sequencer ABI 373XL (Applied Biosystems). Sequences were cleaned from vector sequences and blasted using the non redundant genbank library and also against the *Gallus gallus* genome (http://www.ncbi.nlm.nih.gov/genbank/ and http://www.ncbi.nlm.nih.gov/genome/ or http://www.ensembl.org/Gallus_gallus/). The best 5 results were considered for final identification with a maximum e-value cut-off arbitrarily set up to 2.10^-5^. Corresponding gene identifiers (approved symbols) were used for the annotation of the duck sequences. This heterologous annotation allowed Gene Ontology and pathway analysis using the Ingenuity Pathway Analysis data mining suite (Ingenuity Systems Inc.). Sequences were deposited into the Genbank dbEST database.

### Microarray production

For microarray production, amplifications of cDNA from the 4 subtracted libraries were performed in 96-well plates using bacterial lysates. PCR amplifications were performed using a primer pair corresponding to the flanking adaptor sequences (Clontech) and containing a (CH_2_)_6_-NH2 group at their 5’ end. To ensure quality and quantity amplifications, all PCR products were visualized on 1% agarose gels and then purified using Multiscreen PCR plates (Millipore). Purified products were then quantified on agarose gel and concentrations were adjusted to 200 ng/μl. The cDNA microarray was spotted by the transcriptomic technical platform of BiogenOuest located in Nantes, France, using ROBOT and epoxysilanes coated glass slides.

### Probe preparation and microarray hybridisation

For probe preparation, 96 entire trachea explants were prepared and separated into three groups. Two groups were infected with either a high dose (200 μl of 2×10^7^ TCID50/ml per trachea) of the HPAI H5N1 strain (32 tracheas) or a high dose (200 μl of 2×10^7^ TCID50/ml per trachea) of the LPAI H5N1 strain (32 tracheas). A third group of 32 tracheas was mock-infected (control). Within each groups (infected or not) tracheas were treated individually. At 4 and 8 h p.i., RNA was extracted from 16 out of 32 of each set of tracheas as described in 2.2. RNA was quantified using the Qubit quantitation platform (Invitrogen). For RNA from infected tracheas, four random pools were constructed each with 4 individual RNAs whereas for RNA from mock-infected tracheas only one single reference pool (16 individual RNA) was created to ensure an homogeneous baseline between the different experimental conditions. Quality of RNA in each pool was checked using the Bioanalyzer 2100 platform (Agilent). Probes synthesis was performed with 500 ng of pooled RNA and using the Amino Allyl MessageAmp™ II aRNA Amplification kit (Ambion) and CyDye (Cy3/Cy5) Reactive Dye Pack (Amersham). Two rounds of amplification and a quantity limitation for the 2^nd^ round in order to increase the production of labelled products were performed. Dye swap Cy3/Cy5 was performed between infected and mock-infected samples. Each Cy3- (or Cy5-) labelled sample from infected tracheas was then hybridised with the Cy5- (or Cy3-) labelled reference sample from mock-infected tracheas on our microarray slide. Hybridisations were performed overnight at 42°C in ArrayIt hybridisation chambers (Telechem). Images of the hybridised arrays were acquired by scanning using a Genepix 4000A scanner with the GenePix Pro 5.0 data acquisition and analysis software (Axon Instruments).

### Microarray analysis

Microarray data were processed as previously described [17,56]. Briefly, the raw data were normalised (Lowess) then subjected to a statistical analysis using the significance analysis of microarray (SAM) software to identify differentially expressed genes [57]. SAM software also calculated a false discovery rate (FDR) for each analysis performed. In a first analysis, results obtained with HPAI- or LPAI-infected tracheas were compared to those obtained with mock-infected tracheas at 4 h or 8 h p.i. (SAM one-class analyses). In a second analysis, results obtained with HPAI-infected tracheas were compared to those obtained with LPAI-infected tracheas (SAM two-class analyses). These analyses resulted in a total of 6 datasets (HPAI versus test at 4 and 8 h pi, LPAI versus test at 4 and 8 h pi, HPAI versus LPAI at 4 and 8 h pi) of genes differentially expressed from our different experimental conditions. Results of microarray, SAM analysis and sequencing were combined and used in order to identify implicated cellular pathways by Gene Ontology using the Ingenuity Pathway Analysis software (Ingenuity Systems Inc.). Due to the relative poverty on duck and chicken data in the databases, as compared to the human, mouse or rat data, those analyses were performed by homology with human or mouse genes.

### Real-time PCRs

For the validation of the experimental infection model, in vitro infection of entire trachea, a standard amplification of influenza matrix (M) segment using real-time RT-PCR method was performed [58].

Relative amount of 10 genes of interest as well as control (beta-actin) house-keeping gene expression in HPAI-, LPAI- or mock-infected duck tracheas was analysed by real-time PCR. Each individual RNA sample (see section 2.6) was reverse transcribed using a polydT primer and Superscript II reverse transcriptase (Invitrogen) according to the manufacturer’s protocol. cDNA was subjected to real-time PCR using gene-specific primers (Table 2) and SYBR green technology. Real-time PCR reactions were performed in a final volume of 25 μl with 12.5 μl of SYBR Green PCR Master Mix (Applied Biosystems), 10 μl of cDNA (diluted 1:10) and 200 nM of each forward and reverse primer. Amplifications were performed using the following conditions: 2 min at 50°C, 10 min at 95°C, followed by 45 amplification cycles (15 sec at 95°C and 1 min at 60°C). The uniqueness of the amplification product was assessed by a dissociation stage after the last amplification cycle. Amplifications were carried out in 96-well plates with 3 replicates per sample, using Applied Biosystem 7500 Real-Time PCR Systems and analysed with the Sequence Detection Software. Results were normalized by the control house-keeping gene expression in each sample. Relative amounts of each gene expression were calculated using the R = 2^-ΔΔCT^ equation [59] with gene expression in mock-infected tracheas as relative reference. Two-sample Student t-test was performed to compare gene expression variation between HPAI- and LPAI-infected tracheas, using Systat 9 software (Systat Software Inc., Point Richmond, CA, USA).

**Table 2:** Primer sequences used in real-time quantitative PCR

**Identified gene**	Genbank accession number*	**Primer 1**	**Pimer 2**
B-actin	EF667345	CTTCACCACCACAGCCGAGA	TCGTGGATGCCACAGGACTC
DCN	AF125250	GTGGAGATGATCCATTGATGTAAA	TGTAGCATTCAGCAAATCAAAAAT
DDX3X	XM_002190542	ATATATGTCTTGTGTGCGTGTCCT	TCTAGAAATCTGTCGTGTGCAAAT
EEF1A1	NM_204157	CTTCATTAAGAACATGGTCACTGG	TCCATCTTGTTAACACCAACAATC
FOS	XM_002200534	CTGGGTATCTCCAACTCGTATCTA	ACGTAAGATGGGTCATTGCTAAGT
H-Ras	XM_415752	CCAGGATCCGTTTGTTTCTT	ACAAACTGCGCAAGCTGAAT
IL13	AC233977	AGATAATCTGCTCCATGAGTTTCC	AGCTGCTTTCCATTATTTATCAGC
RABL5	CR406564	CAGTGCAGAGGACACAGAAAATC	ATAATTGACATTTCTTCCTTCTCTCT
RhoA	NM_001245622	GTGTATGATTACTGGCCTTTTTCA	TATCCTGTGAGTGCAGAAAAGGTT
TRIM33	XM_418009	CAGCACAGGTAGCAGAGGAAG	AAGCTGCTCTCAGGACTGCTAA

* Accession numbers correspond to sequences showing the best blast results.

## Competing interests

The authors declare that they have no conflict of interest.

## Authors’ contributions

PM and Véronique Jestin created the original idea of this research and designed the study. PM conducted assays and analysis, data interpretation and drafted the manuscript. CD, AO and HQ performed laboratory methods and preliminary analysis. FXB performed a part of bioinformatics analysis. YB performed statistical analysis, and contributed to the manuscript writing. All authors read and approved the final manuscript.

## Supplementary Material

Additional file 1: Table S1 Ingenuity annotated responding genes to HPAI- or LPAI-infection as compared to control at 8 h post-infection. Click here for file

Additional file 2: Table S2 Ingenuity annotated responding genes to HPAI-infection as compared to LPAI-infection at 8 h post-infection. Click here for file

## References

[B1] Palese P Shaw ML Knipe PMH DM, Griffin DE, Lamb RA, Martin MA, Roizman B, Straus SE Orthomyxoviridae : the viruses and their replication Fields virology 2007 5 Philadelphia: Lippincott-Raven 1647 1689

[B2] Rott R The pathogenic determinant of influenza virus [published erratum appears in Vet Microbiol Apr; 34(4):398] Vet Microbiol 1992 10 303 310 10.1016/0378-1135(92)90058-2 1481363

[B3] Pantin-Jackwood MJ Swayne DE Pathogenesis and pathobiology of avian influenza virus infection in birds Rev Sci Tech 2009 10 113 136 19618622

[B4] FAO-OIE-WHO H5N1 avian influenza: Timeline of major events 2011 Influenza/Human animal interface tripartite note http://www.who.int

[B5] OIE Outbreaks of Highly Pathogenic Avian Influenza (subtype H5N1) in poultry from the end of 2003 to 12 December 2011 2011 Animal health in the world http://www.oie.int

[B6] WHO H5N1 avian influenza: Timeline of major events 2011 H5N1 avian influenza http://www.who.int

[B7] WHO Cumulative number of confirmed human cases for avian influenza A(H5N1) reported to WHO, 2003–2013 2013 H5N1 cumulative table http://www.who.int

[B8] Coleman JR The PB1-F2 protein of Influenza A virus: increasing pathogenicity by disrupting alveolar macrophages Virol J 2007 10 9 10.1186/1743-422X-4-9 17224071 PMC1781424

[B9] Garcia-Sastre A Durbin RK Zheng H Palese P Gertner R Levy DE Durbin JE The role of interferon in influenza virus tissue tropism J Virol 1998 10 8550 8558 9765393 10.1128/jvi.72.11.8550-8558.1998 PMC110265

[B10] Heynisch B Frensing T Heinze K Seitz C Genzel Y Reichl U Differential activation of host cell signalling pathways through infection with two variants of influenza A/Puerto Rico/8/34 (H1N1) in MDCK cells Vaccine 2010 10 8210 8218 10.1016/j.vaccine.2010.07.076 20691654

[B11] Talon J Horvath CM Polley R Basler CF Muster T Palese P Garcia-Sastre A Activation of interferon regulatory factor 3 is inhibited by the influenza A virus NS1 protein J Virol 2000 10 7989 7996 10.1128/JVI.74.17.7989-7996.2000 10933707 PMC112330

[B12] Watanabe T Watanabe S Kawaoka Y Cellular networks involved in the influenza virus life cycle Cell Host Microbe 2010 10 427 439 10.1016/j.chom.2010.05.008 20542247 PMC3167038

[B13] Zamarin D Garcia-Sastre A Xiao X Wang R Palese P Influenza virus PB1-F2 protein induces cell death through mitochondrial ANT3 and VDAC1 PLoS Pathog 2005 10 e4 10.1371/journal.ppat.0010004 16201016 PMC1238739

[B14] Alkhalil A Hammamieh R Hardick J Ichou MA Jett M Ibrahim S Gene expression profiling of monkeypox virus-infected cells reveals novel interfaces for host-virus interactions Virol J 2010 10 173 10.1186/1743-422X-7-173 20667104 PMC2920256

[B15] Almeida PE Weber PS Burton JL Tempelman RJ Steibel JP Zanella AJ Gene expression profiling of peripheral mononuclear cells in lame dairy cows with foot lesions Vet Immunol Immunopathol 2007 10 234 245 10.1016/j.vetimm.2007.06.028 17675248

[B16] Mo XY Ma W Zhang Y Zhao H Deng Y Yuan W Wang Y Li Y Zhu C Liu M Wu X Microarray analyses of differentially expressed human genes and biological processes in ECV304 cells infected with rubella virus J Med Virol 2007 10 1783 1791 10.1002/jmv.20942 17854033

[B17] Renson P Blanchard Y Le-Dimna M Felix H Cariolet R Jestin A Le-Potier MF Acute induction of cell death-related IFN stimulated genes (ISG) differentiates highly from moderately virulent CSFV strains Vet Res 2010 10 7 10.1051/vetres/2009055 19793538 PMC2775166

[B18] Tong HH Long JP Li D DeMaria TF Alteration of gene expression in human middle ear epithelial cells induced by influenza A virus and its implication for the pathogenesis of otitis media Microb Pathog 2004 10 193 204 10.1016/j.micpath.2004.06.012 15458780

[B19] Yu C Boon D McDonald SL Myers TG Tomioka K Nguyen H Engle RE Govindarajan S Emerson SU Purcell RH Pathogenesis of hepatitis E virus and hepatitis C virus in chimpanzees: similarities and differences J Virol 2010 10 11264 11278 10.1128/JVI.01205-10 20739520 PMC2953165

[B20] Zornetzer GA Frieman MB Rosenzweig E Korth MJ Page C Baric RS Katze MG Transcriptomic analysis reveals a mechanism for a prefibrotic phenotype in STAT1 knockout mice during severe acute respiratory syndrome coronavirus infection J Virol 2010 10 11297 11309 10.1128/JVI.01130-10 20702617 PMC2953159

[B21] Suzuki K Okada H Itoh T Tada T Mase M Nakamura K Kubo M Tsukamoto K Association of increased pathogenicity of Asian H5N1 highly pathogenic avian influenza viruses in chickens with highly efficient viral replication accompanied by early destruction of innate immune responses J Virol 2009 10 7475 7486 10.1128/JVI.01434-08 19457987 PMC2708648

[B22] Wang Y Brahmakshatriya V Zhu H Lupiani B Reddy SM Yoon BJ Gunaratne PH Kim JH Chen R Wang J Zhou H Identification of differentially expressed miRNAs in chicken lung and trachea with avian influenza virus infection by a deep sequencing approach BMC Genomics 2009 10 512 10.1186/1471-2164-10-512 19891781 PMC2777939

[B23] Xing Z Cardona CJ Li J Dao N Tran T Andrada J Modulation of the immune responses in chickens by low-pathogenicity avian influenza virus H9N2 J Gen Virol 2008 10 1288 1299 10.1099/vir.0.83362-0 18420808

[B24] Dar A Munir S Vishwanathan S Manuja A Griebel P Tikoo S Townsend H Potter A Kapur V Babiuk LA Transcriptional analysis of avian embryonic tissues following infection with avian infectious bronchitis virus Virus Res 2005 10 41 55 10.1016/j.virusres.2005.01.006 15845254 PMC7114260

[B25] Li X Chiang HI Zhu J Dowd SE Zhou H Characterization of a newly developed chicken 44K Agilent microarray BMC Genomics 2008 10 60 10.1186/1471-2164-9-60 18237426 PMC2262898

[B26] Munir S Sharma JM Kapur V Transcriptional response of avian cells to infection with Newcastle disease virus Virus Res 2005 10 103 108 10.1016/j.virusres.2004.07.001 15567039

[B27] Reemers SS Groot Koerkamp MJ Holstege FC Van-Eden W Vervelde L Cellular host transcriptional responses to influenza A virus in chicken tracheal organ cultures differ from responses in in vivo infected trachea Vet Immunol Immunopathol 2009 10 91 100 10.1016/j.vetimm.2009.04.021 19447504

[B28] Reemers SS Van-Leenen D Koerkamp MJ Van-Haarlem D van de-Haar P Van-Eden W Vervelde L Early host responses to avian influenza A virus are prolonged and enhanced at transcriptional level depending on maturation of the immune system Mol Immunol 2010 10 1675 1685 10.1016/j.molimm.2010.03.008 20382427

[B29] Sarmento L Afonso CL Estevez C Wasilenko J Pantin-Jackwood M Differential host gene expression in cells infected with highly pathogenic H5N1 avian influenza viruses Vet Immunol Immunopathol 2008 10 291 302 10.1016/j.vetimm.2008.05.021 18617273

[B30] Zhang W Li H Cheng G Hu S Li Z Bi D Avian influenza virus infection induces differential expression of genes in chicken kidney Res Vet Sci 2008 10 374 381 10.1016/j.rvsc.2007.05.015 17692877

[B31] Huang YH Li N Burt DW Wu F Genomic research and applications in the duck (Anas platyrhynchos) Worlds Poult Sci J 2008 10 329 341

[B32] Huang Y Li Y Burt DW Chen H Zhang Y Qian W Kim H Gan S Zhao Y Li J The duck genome and transcriptome provide insight into an avian influenza virus reservoir species Nat Genet 2013 10 776 783 10.1038/ng.2657 23749191 PMC4003391

[B33] Fleming-Canepa X Brusnyk C Aldridge JR Ross KL Moon D Wang D Xia J Barber MR Webster RG Magor KE Expression of duck CCL19 and CCL21 and CCR7 receptor in lymphoid and influenza-infected tissues Mol Immunol 2011 10 1950 1957 10.1016/j.molimm.2011.05.025 21704378 PMC3163774

[B34] Vanderven HA Petkau K Ryan-Jean KE Aldridge JR Jr Webster RG Magor KE Avian influenza rapidly induces antiviral genes in duck lung and intestine Mol Immunol 2012 10 316 324 10.1016/j.molimm.2012.03.034 22534314 PMC3358531

[B35] Adams SC Xing Z Li J Cardona CJ Immune-related gene expression in response to H11N9 low pathogenic avian influenza virus infection in chicken and Pekin duck peripheral blood mononuclear cells Mol Immunol 2009 10 1744 1749 10.1016/j.molimm.2009.01.025 19250679

[B36] Cagle C To TL Nguyen T Wasilenko J Adams SC Cardona CJ Spackman E Suarez DL Pantin-Jackwood MJ Pekin and Muscovy ducks respond differently to vaccination with a H5N1 highly pathogenic avian influenza (HPAI) commercial inactivated vaccine Vaccine 2011 10 6549 6557 10.1016/j.vaccine.2011.07.004 21771626

[B37] Phuong do Q Dung NT Jorgensen PH Handberg KJ Vinh NT Christensen JP Susceptibility of Muscovy (Cairina Moschata) and mallard ducks (Anas Platyrhynchos) to experimental infections by different genotypes of H5N1 avian influenza viruses Vet Microbiol 2010 10 168 174 20943331 10.1016/j.vetmic.2010.09.007

[B38] Dios S Poisa-Beiro L Figueras A Novoa B Suppression subtraction hybridization (SSH) and macroarray techniques reveal differential gene expression profiles in brain of sea bream infected with nodavirus Mol Immunol 2007 10 2195 2204 10.1016/j.molimm.2006.11.017 17188359

[B39] Fang Q Wang L Zhu J Li Y Song Q Stanley DW Akhtar ZR Ye G Expression of immune-response genes in lepidopteran host is suppressed by venom from an endoparasitoid. Pteromalus puparum BMC Genomics 2010 10 484 10.1186/1471-2164-11-484 20813030 PMC2996980

[B40] Hu TX Yu M Zhao J Comparative transcriptional profiling analysis of the two daughter cells from tobacco zygote reveals the transcriptome differences in the apical and basal cells BMC Plant Biol 2010 10 167 10.1186/1471-2229-10-167 20699003 PMC3095300

[B41] Munir S Singh S Kaur K Kapur V Suppression subtractive hybridization coupled with microarray analysis to examine differential expression of genes in virus infected cells Biol Proced Online 2004 10 94 104 10.1251/bpo77 15181476 PMC420231

[B42] Lake J Gravel C Koko GK Robert C Vandenberg GW Combining suppressive subtractive hybridization and cDNA microarrays to identify dietary phosphorus-responsive genes of the rainbow trout (Oncorhynchus mykiss) kidney Comp Biochem Physiol Part D Genomics Proteomics 2010 10 24 35 10.1016/j.cbd.2009.09.002 20374939

[B43] Pan YS Lee YS Lee YL Lee WC Hsieh SY Differentially profiling the low-expression transcriptomes of human hepatoma using a novel SSH/microarray approach BMC Genomics 2006 10 131 10.1186/1471-2164-7-131 16737534 PMC1522022

[B44] Zou X Jiang Y Liu L Zhang Z Zheng Y Identification of transcriptome induced in roots of maize seedlings at the late stage of waterlogging BMC Plant Biol 2010 10 189 10.1186/1471-2229-10-189 20738849 PMC2956539

[B45] Hsu AC Barr I Hansbro PM Wark PA Human influenza is more effective than avian influenza at antiviral suppression in airway cells Am J Respir Cell Mol Biol 2010 10 906 913 20705938 10.1165/rcmb.2010-0157OC PMC3135850

[B46] Iwai A Shiozaki T Kawai T Akira S Kawaoka Y Takada A Kida H Miyazaki T Influenza A virus polymerase inhibits type I interferon induction by binding to interferon beta promoter stimulator 1 J Biol Chem 2010 10 32064 32074 10.1074/jbc.M110.112458 20699220 PMC2952208

[B47] Liu H Golebiewski L Dow EC Krug RM Javier RT Rice AP The ESEV PDZ-binding motif of the avian influenza A virus NS1 protein protects infected cells from apoptosis by directly targeting Scribble J Virol 2010 10 11164 11174 10.1128/JVI.01278-10 20702615 PMC2953166

[B48] Wang S Quang Le T Chida J Cisse Y Yano M Kido H Mechanisms of matrix metalloproteinase-9 upregulation and tissue destruction in various organs in influenza A virus infection J Med Invest 2010 10 26 34 10.2152/jmi.57.26 20299740

[B49] Hale BG Randall RE Ortin J Jackson D The multifunctional NS1 protein of influenza A viruses J Gen Virol 2008 10 2359 2376 10.1099/vir.0.2008/004606-0 18796704

[B50] Wu Y Yoder A Chemokine coreceptor signaling in HIV-1 infection and pathogenesis PLoS Pathog 2009 10 e1000520 10.1371/journal.ppat.1000520 20041213 PMC2790611

[B51] Kang SM Lim S Won SJ Shin YJ Lim YS Ahn BY Hwang SB c-Fos regulates hepatitis C virus propagation FEBS Lett 2011 10 3236 3244 10.1016/j.febslet.2011.08.041 21920361

[B52] Forrester NA Patel RN Speiseder T Groitl P Sedgwick GG Shimwell NJ Seed RI Catnaigh PO McCabe CJ Stewart GS Adenovirus E4orf3 targets transcriptional intermediary factor 1gamma for proteasome-dependent degradation during infection J Virol 2012 10 3167 3179 10.1128/JVI.06583-11 22205733 PMC3302322

[B53] Cilloniz C Shinya K Peng X Korth MJ Proll SC Aicher LD Carter VS Chang JH Kobasa D Feldmann F Lethal influenza virus infection in macaques is associated with early dysregulation of inflammatory related genes PLoS Pathog 2009 10 e1000604 10.1371/journal.ppat.1000604 19798428 PMC2745659

[B54] Briand FX Le-Gall-Reculé G Guillou-Cloarec C Ogor K Jestin V Phylogeny and genotyping of recent avian low-pathogenic H5 subtype influenza viruses from French ducks J Gen Virol 2010 10 960 970 10.1099/vir.0.016733-0 20016038

[B55] Gall-Recule GL Briand FX Schmitz A Guionie O Massin P Jestin V Double introduction of highly pathogenic H5N1 avian influenza virus into France in early 2006 Avian Pathol 2008 10 15 23 10.1080/03079450701774835 18202945

[B56] Blanchard Y Le-Meur N Le-Cunff M Blanchard P Leger J Jestin A Cellular gene expression survey of PseudoRabies Virus (PRV) infected Human Embryonic Kidney cells (HEK-293) Vet Res 2006 10 705 723 10.1051/vetres:2006027 16820135

[B57] Tusher VG Tibshirani R Chu G Significance analysis of microarrays applied to the ionizing radiation response Proc Natl Acad Sci USA 2001 10 5116 5121 10.1073/pnas.091062498 11309499 PMC33173

[B58] Spackman E Senne DA Myers TJ Bulaga LL Garber LP Perdue ML Lohman K Daum LT Suarez DL Development of a real-time reverse transcriptase PCR assay for type A influenza virus and the avian H5 and H7 hemagglutinin subtypes J Clin Microbiol 2002 10 3256 3260 10.1128/JCM.40.9.3256-3260.2002 12202562 PMC130722

[B59] Livak KJ Schmittgen TD Analysis of relative gene expression data using real-time quantitative PCR and the 2(−Delta Delta C(T)) Method Methods 2001 10 402 408 10.1006/meth.2001.1262 11846609

