# Supplementary material for: Differential cellular gene expression in duck trachea infected with a highly or low pathogenic H5N1 avian influenza virus
Source: Virol J. 2013 Sep 10;10:279. doi: 10.1186/1743-422X-10-279 (PMC3848638; doi:10.1186/1743-422X-10-279)

**Additional file 2: Table S2** Ingenuity annotated responding genes to HPAI-infection as compared to LPAI-infection at 8h post-infection.


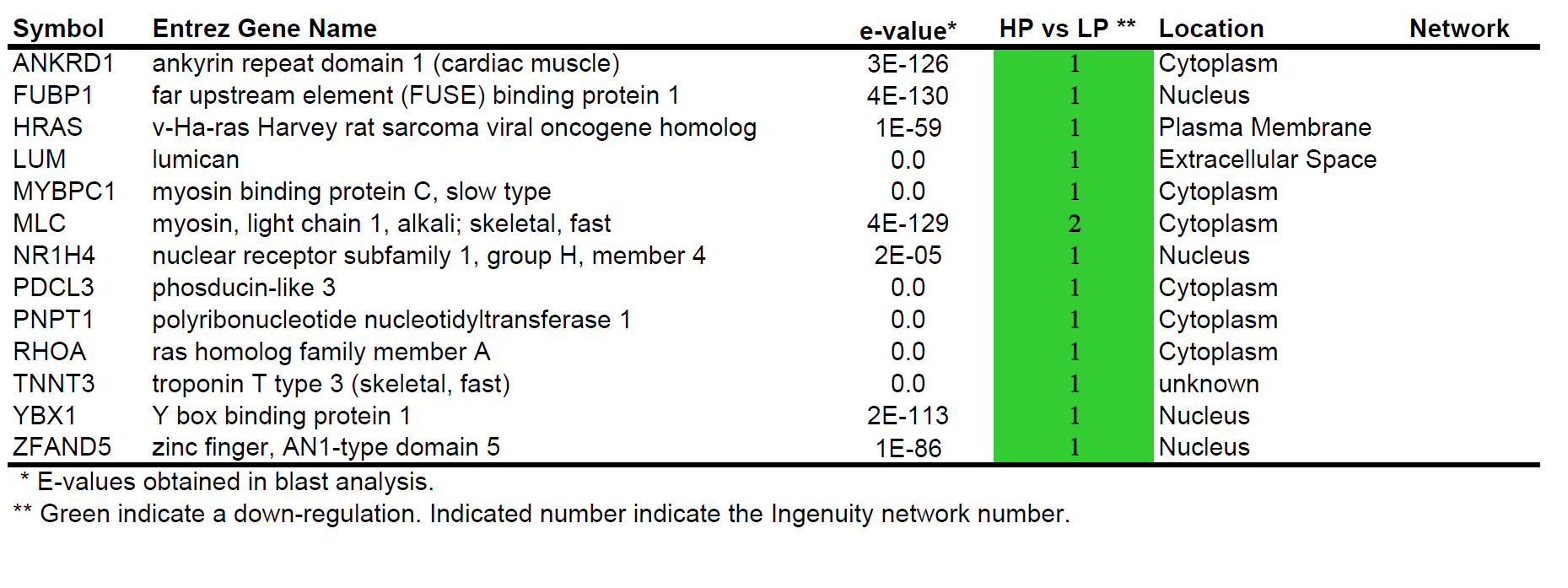

Supplement: Additional file 2: Table S2 — Ingenuity annotated responding genes to HPAI-infection as compared to LPAI-infection at 8 h post-infection. [file 1743-422X-10-279-S2.doc]
